# The relationship between system inflammation response index and coronary heart disease: a cross-sectional study (NHANES 2007–2016)

**DOI:** 10.3389/fcvm.2024.1439913

**Published:** 2024-08-12

**Authors:** Tian Yang Zhang, Hai long Chen, Yanyu Shi, Ying Jin, Yuan Zhang, Ying Chen

**Affiliations:** ^1^ College of Chinese Medicine, Changchun University of Chinese Medicine, Changchun, China; ^2^ Department of Chronic Disease Clinic, Changchun NanGuan District Hospital, Changchun, China; ^3^ Department of Cardiology, Affiliated Hospital of Changchun University of Chinese Medicine, Changchun, China

**Keywords:** system inflammation response index, inflammation, coronary heart disease, NHANES, cross-sectional study

## Abstract

**Background:**

Coronary heart disease (CHD) is one of the common chronic diseases in clinical practice, often accompanied by inflammatory reactions. In recent years, the system inflammation response index (SIRI) has aroused researchers’ interest as a novel inflammatory biomarker. This study aims to explore the relationship between the SIRI and CHD through the National Health and Nutrition Examination Survey (NHANES) database.

**Methods:**

We conducted a cross-sectional study and analyzed participants aged 40 and above with complete data from the NHANES survey years 2007–2016. Logistic regression analysis was used in this study to explore the relationship between the risk of CHD and SIRI. Stratified subgroup analysis was conducted based on age, gender, race, education level, body mass index (BMI), smoking status, drinking, hypertension, diabetes and angina pectoris to evaluate the relationship between SIRI and CHD in different populations. Additionally, restricted cubic spline (RCS) analysis was employed to investigate whether there is a nonlinear association between SIRI and CHD.

**Results:**

A total of 6374 eligible participants were included, among whom 387 were diagnosed with CHD. The SIRI levels in the CHD group were significantly higher than those in the non-CHD group. After adjusting for potential confounders, an elevated SIRI level was associated with an increased risk of CHD, with an odds ratio of 1.12, 95% CI: (1.03, 1.22), *P *= 0.008. Subgroup analysis results indicated a significant interaction between SIRI and CHD among genders (*P* for interaction <0.05), especially in females. In contrast, no significant interaction was observed among age, race, education level, BMI, smoking status, drinking, hypertension, diabetes and angina pectoris (*P* for interaction >0.05). The RCS analysis showed a significant linear relationship between SIRI and CHD (*P* for non-linearity >0.05), with an inflection point at 2.86.

**Conclusion:**

Our study indicates that an elevated system inflammation response index is associated with a higher risk of CHD. Particularly among women.

## 1 Introduction

Coronary heart disease is a common cardiovascular disease in clinical practice and a leading cause of global morbidity and mortality (1). According to NHANES data from 2017 to 2020, an estimated 20.5 million Americans are affected by CHD. The prevalence of CHD is higher in men than in women (2). In the population with heart disease in the United States, CHD accounts for the highest proportion of deaths (3). Every 1 out of 6 deaths is due to CHD (4). Expenditure related to CHD has significantly increased in developed countries, posing a heavy burden on healthcare systems (2, 5).

The pathogenesis of CHD mainly involves atherosclerosis and coronary artery spasm, among which the formation and development of atherosclerosis are the most important factors (6). Additionally, vascular endothelial injury, lipid metabolism disorders, and inflammatory reactions can promote the occurrence and development of coronary atherosclerosis, leading to CHD (7, 8). The formation process of coronary atherosclerosis is complex. Early vascular endothelial dysfunction, lipid deposition, and later stages lead to the formation of calcified plaques. Among them, vulnerable plaques are unstable and prone to thrombosis, which is closely related to myocardial infarction (9). Hypertension, smoking, diabetes, dyslipidemia, and inflammatory reactions are high-risk factors for CHD (10, 11). Among all risk factors, the role of inflammation in the development of CHD has always been a focus of researchers (12, 13). The SIRI, as a new nonspecific inflammation marker, integrates neutrophils, monocytes, and lymphocytes, reflecting systemic inflammation, and is cost-effective and easy to detect (14). In recent years, SIRI has emerged as a new indicator for predicting the risk of inflammatory diseases. The study by Cai et al. found that elevated SIRI levels were significantly associated with the risk of stroke and its subtypes in elderly hypertensive patients, suggesting that SIRI may serve as a potential indicator for predicting stroke risk (15). Ma et al. discovered a potential association between SIRI and bone mineral density, osteoporosis, and future fracture risk in elderly hypertensive patients. However, further studies are needed to confirm these findings (16). Currently, there are still few studies on the use of SIRI in CHD. Considering that individuals aged 40 and above are more prone to CHD. Therefore, this study aims to explore the potential correlation between SIRI and the risk of CHD in middle-aged and elderly populations using large-sample data from the NHANES, which may contribute to the prevention and treatment of CHD.

## 2 Methods

### 2.1 Data sources

Our data is sourced from NHANES, a population-based cross-sectional survey conducted by the Centers for Disease Control and Prevention (CDC) to assess adults’ and children's health and nutritional status. The research plan is implemented by a team of professional health surveyors, medical technicians, and doctors. The NHANES database contains demographic data, dietary data, examination data, laboratory data, and questionnaire data. It is updated every two years. NHANES participants provide informed consent, and the National Center for Health Statistics (NCHS) Ethics Review Board approves the study. Representative survey participants were selected using a “stratified multi-stage probability sampling” method. Each year, approximately 5,000 participants from various geographic regions and socioeconomic backgrounds are randomly selected to participate in the survey. In terms of quality control measures for laboratory measurements, collected biological samples are transported and stored under strictly controlled conditions to prevent contamination or deterioration. Standardized processing and analytical procedures are used to ensure consistency and comparability of results. Each laboratory regularly uses standard substances for calibration to monitor the accuracy and precision of the instruments. In summary, NHANES ensures high quality and reliability in its data collection and laboratory measurements through stringent quality control measures, making it a vital data source for nutrition and health research. For detailed methods, please refer to the NHANES website (https://www.cdc.gov/nchs/nhanes/index.htm). This study follows the reporting guidelines of the Strengthening the Reporting of Observational Studies in Epidemiology (STROBE) for cross-sectional studies.

### 2.2 Participant selection and process

**Figure 1:**
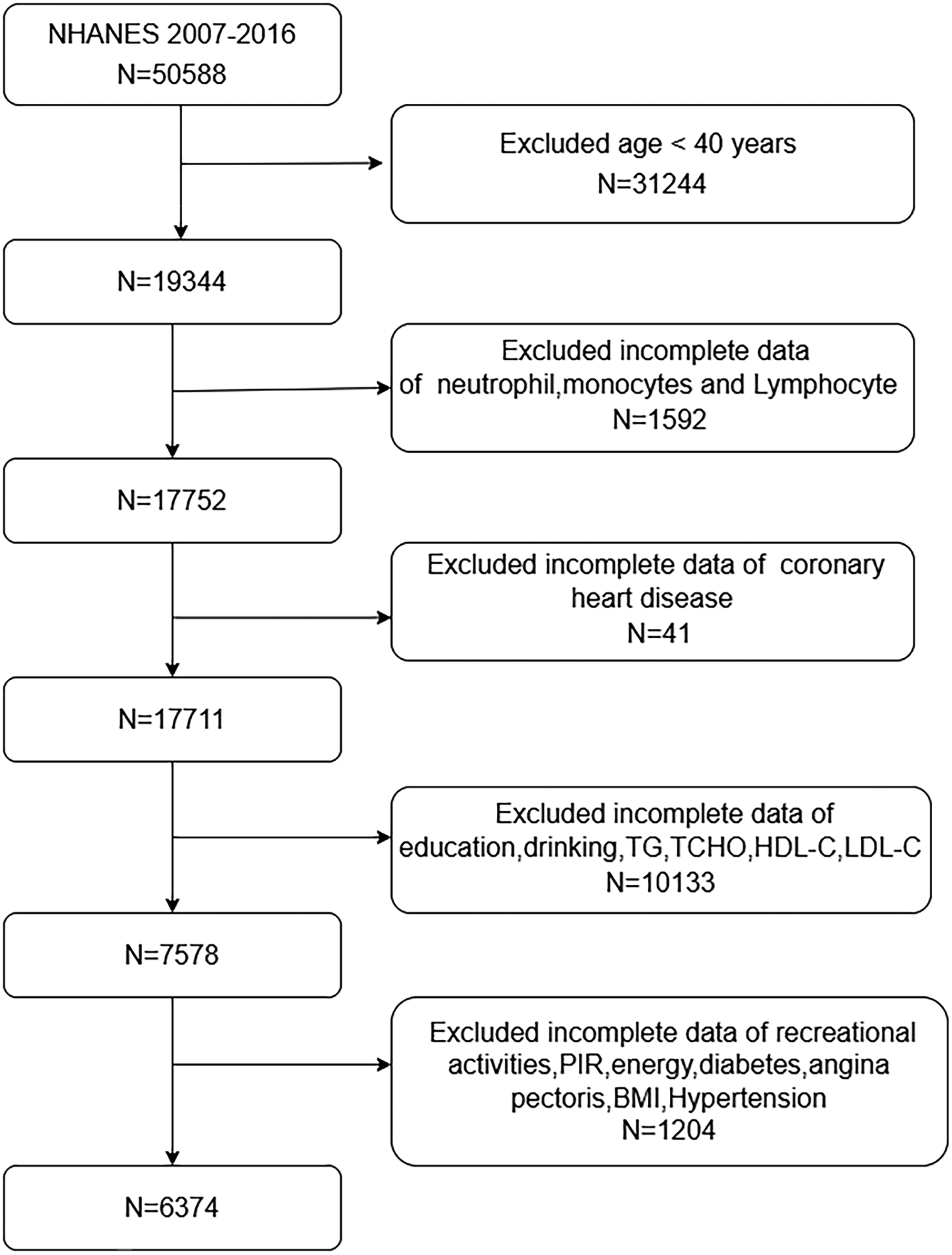
Flow diagram of participants screened from the national health and nutrition examination survey (NHANES) 2007–2016.

We used data from 2007 to 2016 to select participants. We initially screened 50,588 participants, with the specific exclusion criteria as follows: Exclude participants < 40 years old (*n* = 31,244); Exclude participants missing monocytes, neutrophil and lymphocyte counts (*n* = 1,592); Exclude participants lacking CHD data (*n* = 41); Exclude participants missing education, drinking, TG, TCHO, HDL-C, LDL-C data (*n* = 10,133); Exclude participants lacking recreational activities, PIR, energy, diabetes, angina pectoris, BMI, hypertension data (*n* = 1,204). A total of 6,374 eligible participants were included (Figure 1).

### 2.3 Detection and definition of SIRI

Fasting venous blood samples were collected in the morning at the Mobile Examination Center (MEC) of NHANES after an overnight fast. The Beckman Coulter D × H 800 instrument at the MEC was used for complete blood cell counts. The definition of SIRI is as follows: SIRI = M × N/l, where M, N, and L represent the counts of monocytes, neutrophils, and lymphocytes, respectively (17).

### 2.4 Definition of coronary heart disease, diabetes, and hypertension

In the questionnaire, participants were asked, “Ever told you had coronary heart disease.” Those who answered “yes” were classified as having coronary heart disease (18).

Diagnostic criteria for diabetes: (1) Participants were informed of having diabetes. (2) Fasting blood glucose (mmol/L) ≥ 7.0 mmol/l. (3) Symptoms of diabetes combined with random blood glucose ≥ 11.1 mmol/l. (4) Glycated hemoglobin A1c (HbA1c) (%) ≥ 6.5% (19).

The diagnostic criteria for hypertension: In the questionnaire, all participants were asked, “Ever told you had high blood pressure?” with response options of “Yes” or “No.” Participants who responded “Yes” were defined as having high blood pressure, while those who answered “No” were defined as not having high blood pressure.

### 2.5 Covariates

The covariates included demographic data, Examination Data, and laboratory data. Specifically, they are as follows: age (40–59 years and ≥60 years), gender (male and female), race (Mexican American, Other Hispanic, Non-Hispanic White, Non-Hispanic Black, and Other), education level (above/below high school), body mass index (BMI) categorized into three groups: normal, overweight, and obese (<25 kg/m^2^, 25–29.9 kg/m^2^, ≥ 30 kg/m^2^), and smoking status (Never, Former, Current). Participants were asked if they had smoked 100 cigarettes in their lifetime and if they currently smoked to determine current and former smokers. Those who had smoked fewer than 100 cigarettes in their lifetime were defined as never smokers. Former smokers were defined as participants who did not currently smoke but had smoked 100 cigarettes in the past. The activity was defined as any moderate-intensity exercise, fitness, or recreational activity leading to a slight increase in breathing or heart rate-such as brisk walking, bicycling, swimming, or volleyball for at least ten consecutive minutes weekly. Drinkers were defined as those who consumed at least 12 drinks of alcohol in any given year. Additionally, we included variables such as diabetes, angina pectoris, hypertension, PIR, energy, TCHO, TG, LDL-C, and HDL-C. All covariates were obtained from the NHANES database.

### 2.6 Statistical analysis

DecisionLinnc1.0 software was employed for data analysis (20). Categorical variables were expressed as percentages, while continuous variables were expressed as means ± standard deviations. In this study, we used logistic regression analysis because it is advantageous in handling binary outcome variables while adjusting for potential confounders. Using RCS curves can more accurately describe the relationship between continuous variables and the risk of outcomes. SIRI was categorized into quartiles (Q1-Q4), with the Q1 group as the logistic regression reference group. Model 1 was not adjusted for covariates. Model 2 was adjusted for age, sex, and race. In Model 3, we adjusted for age, gender, race, education level, BMI, smoking status, diabetes, drinking, angina pectoris, hypertension, recreational activities, PIR, energy, TCHO, TG, LDL-C, HDL-C. We also conducted subgroup analyses in different populations to evaluate differences among them. Additionally, we used RCS to explore the nonlinear relationship between SIRI and the risk of CHD. A *p*-value < 0.05 was considered statistically significant.

## 3 Results

### 3.1 The characteristics of the participants

A total of 6,374 participants with complete data were included in this study (Figure 1). Among them, 5,987 were non-CHD participants, and 387 were CHD participants. Females accounted for 33.07%, while males accounted for 66.93%. The mean SIRI ± SD was 1.23 ± 0.99. 6.07% of patients had coronary artery lesions. Compared with the non-CHD group, the CHD group had older age (*P *< 0.001), predominantly male (*P* < 0.001), a higher proportion of Non-Hispanic Whites (*P* < 0.001), a higher education level (*P* < 0.05), and a higher proportion of smokers (*P* < 0.001). The CHD patients had a higher proportion of hypertension and diabetes compared to the non-CHD patients (*P* < 0.001). There were statistically significant differences in TCHO, TG, LDL-C, and HDL-C between the two groups (*P* < 0.05) see Table 1 for details.

**Table 1:** Baseline characteristics of the study participants.

Variable Names	Overall	Non-CHD	CHD	*P*
*n*	6,374	5,987	387	
Age (%)				
40–59	3,192 (50.08)	3,140 (52.45)	52 (13.44)	<0.001
≥60	3,182 (49.92)	2,847 (47.55)	335 (86.56)	
Gender (%)				
Male	3,107 (48.74)	2,848 (47.57)	259 (66.93)	<0.001
Female	3,267 (51.26)	3,139 (52.43)	128 (33.07)	
Race (%)				
Mexican American	876 (13.74)	843 (14.08)	33 (8.53)	<0.001
Other Hispanic	661 (10.37)	626 (10.46)	35 (9.04)	
Non-Hispanic White	3,119 (48.93)	2,863 (47.82)	256 (66.15)	
Non-Hispanic Black	1,234 (19.36)	1,191 (19.89)	43 (11.11)	
Other race	484 (7.59)	464 (7.75)	20 (5.17)	
Education level (%)				
<High school	1,658 (26.01)	1,536 (25.66)	122 (31.52)	0.013
≥High school	4,716 (73.99)	4,451 (74.34)	265 (68.48)	
BMI (%)				
<25	1,640 (25.73)	1,548 (25.86)	92 (23.77)	0.257
25–29.9	2,235 (35.06)	2,107 (35.19)	128 (33.07)	
≥30	2,499 (39.21)	2,332 (38.95)	167 (43.15)	
Smoking status (%)				
Never	3,255 (51.07)	3,095 (51.70)	160 (41.34)	<0.001
Former	1,952 (30.62)	1,790 (29.90)	162 (41.86)	
Current	1,167 (18.31)	1,102 (18.41)	65 (16.80)	
Diabetes (%)				
Yes	1,156 (18.14)	1,021 (17.05)	135 (34.88)	<0.001
No	5,218 (81.86)	4,966 (82.95)	252 (65.12)	
Drinking (%)				
Yes	4,456 (69.91)	4,179 (69.80)	277 (71.58)	0.496
No	1,918 (30.09)	1,808 (30.20)	110 (28.42)	
Angina pectoris (%)				
Yes	224 (3.51)	106 (1.77)	118 (30.49)	<0.001
No	6,150 (96.49)	5,881 (98.23)	269 (69.51)	
Hypertension (%)				
Yes	3,064 (48.07)	2,760 (46.10)	304 (78.55)	<0.001
No	3,310 (51.93)	3,227 (53.90)	83 (21.45)	
Recreational activities (%)				
Yes	2,433 (38.17)	2,294 (38.32)	139 (35.92)	0.375
No	3,941 (61.83)	3,693 (61.68)	248 (64.08)	
PIR	2.64 ± 1.63	2.64 ± 1.63	2.50 ± 1.54	0.103
Energy (kcal)	1,987.65 ± 902.26	1,994.43 ± 903.07	1,882.64 ± 884.18	0.018
TCHO (mg/dl)	196.59 ± 41.64	198.29 ± 40.89	170.34 ± 44.32	<0.001
TG (mg/dl)	123.67 ± 65.46	123.15 ± 64.89	131.80 ± 73.35	0.012
LDL-C (mg/dl)	116.77 ± 36.39	118.27 ± 35.87	93.53 ± 36.58	<0.001
HDL-C(mg/dl)	55.09 ± 16.78	55.39 ± 16.77	50.45 ± 16.22	<0.001
SIRI	1.23 ± 0.99	1.20 ± 0.95	1.78 ± 1.40	<0.001

Mean ± SD for continuous variables and % for categorical variables. CHD, coronary heart disease; BMI, body mass index; PIR, poverty income ratio; TCHO, total cholesterol; TG, triglyceride; LDL-C, low-density lipoprotein cholesterol; HDL-C, high-density lipoprotein cholesterol; SIRI, systemic inflammatory response index.

### 3.2 The relationship between SIRI and CHD

In the overall population, univariate logistic regression results showed a positive correlation between SIRI and CHD (OR: 1.43, 95% CI: 1.32, 1.54). After adjusting for age, gender, and race in Model 2, this relationship remained (OR: 1.24, 95% CI: 1.14, 1.35). In Model 3, after adjusting for all covariates, SIRI remained significantly associated with CHD incidence (OR: 1.12, 95% CI: 1.03–1.22, *P* = 0.008). When SIRI was categorized into quartiles, with Q1 as the reference, the OR for Q4 was significantly higher than that for Q1 (OR: 4.50, 95% CI: 3.22–6.43, *P* < 0.001).

Following complete adjustment for all covariates, Patients in the highest quartile of SIRI have a risk of developing the disease that is more than one time higher than those in the lowest quartile (OR: 1.78, 95% CI: 1.20–2.68, *P* = 0.005). Detailed results are shown in Table 2.

**Table 2:** The association between SIRI levels and prevalence of CHD by logistic regression analyses.

		Model 1		Model 2		Model 3	
		OR (95% CI)	*P*-value	OR (95% CI)	*P*-value	OR (95% CI)	*P*-value
SIRI		1.43 (1.32, 1.54)	<0.001	1.24 (1.14, 1.35)	<0.001	1.12 (1.03, 1.22)	0.008
SIRI (quartile)							
Q1	0.48 ± 0.13	Reference		Reference		Reference	
Q2	0.83 ± 0.09	1.85 (1.27, 2.74)	0.002	1.48 (1.00, 2.21)	0.051	1.39 (0.92, 2.14)	0.127
Q3	1.20 ± 0.14	2.37 (1.65, 3.46)	<0.001	1.61 (1.11, 2.39)	0.015	1.35 (0.89, 2.06)	0.160
Q4	2.41 ± 1.33	4.50 (3.22, 6.43)	<0.001	2.45 (1.71, 3.57)	<0.001	1.78 (1.20, 2.68)	0.005
*P* for trend		<0.001		<0.001		0.005	

95% CI, 95% confidence interval.

Model 1: no covariates were adjusted.

Model 2: adjusted for age, gender, race.

Model 3: adjusted for age, gender, race, education level, BMI, smoking status, diabetes, drinking, angina pectoris, hypertension, recreational activities, PIR, energy, TCHO, TG, LDL-C, HDL-C.

### 3.3 Subgroup analysis

**Figure 2:**
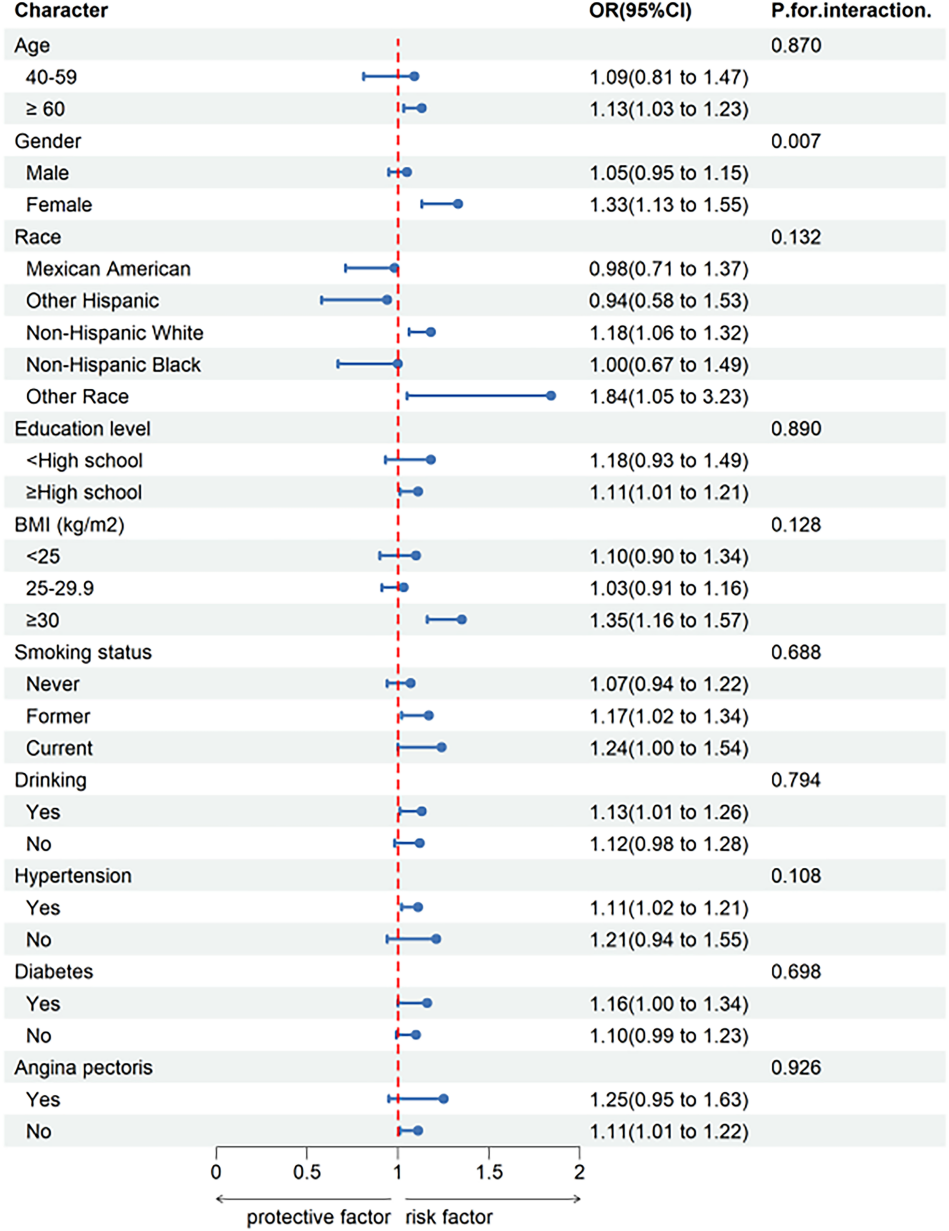
Subgroup analysis for the association between SIRI and coronary heart disease.

To validate the stability of the relationship between SIRI and CHD in different subgroups, subgroup analyses were conducted based on Model 3. The results are shown in Figure 2. There was a significant interaction between SIRI and CHD by gender (*P* for interaction < 0.05), indicating that the relationship between increased SIRI and CHD risk is more pronounced in females than males. However, interactions were not significant for age, race, education level, BMI, smoking status, drinking, hypertension, angina pectoris and diabetes (*P* for interaction > 0.05).

### 3.4 Linear association between SIRI and CHD

**Figure 3:**
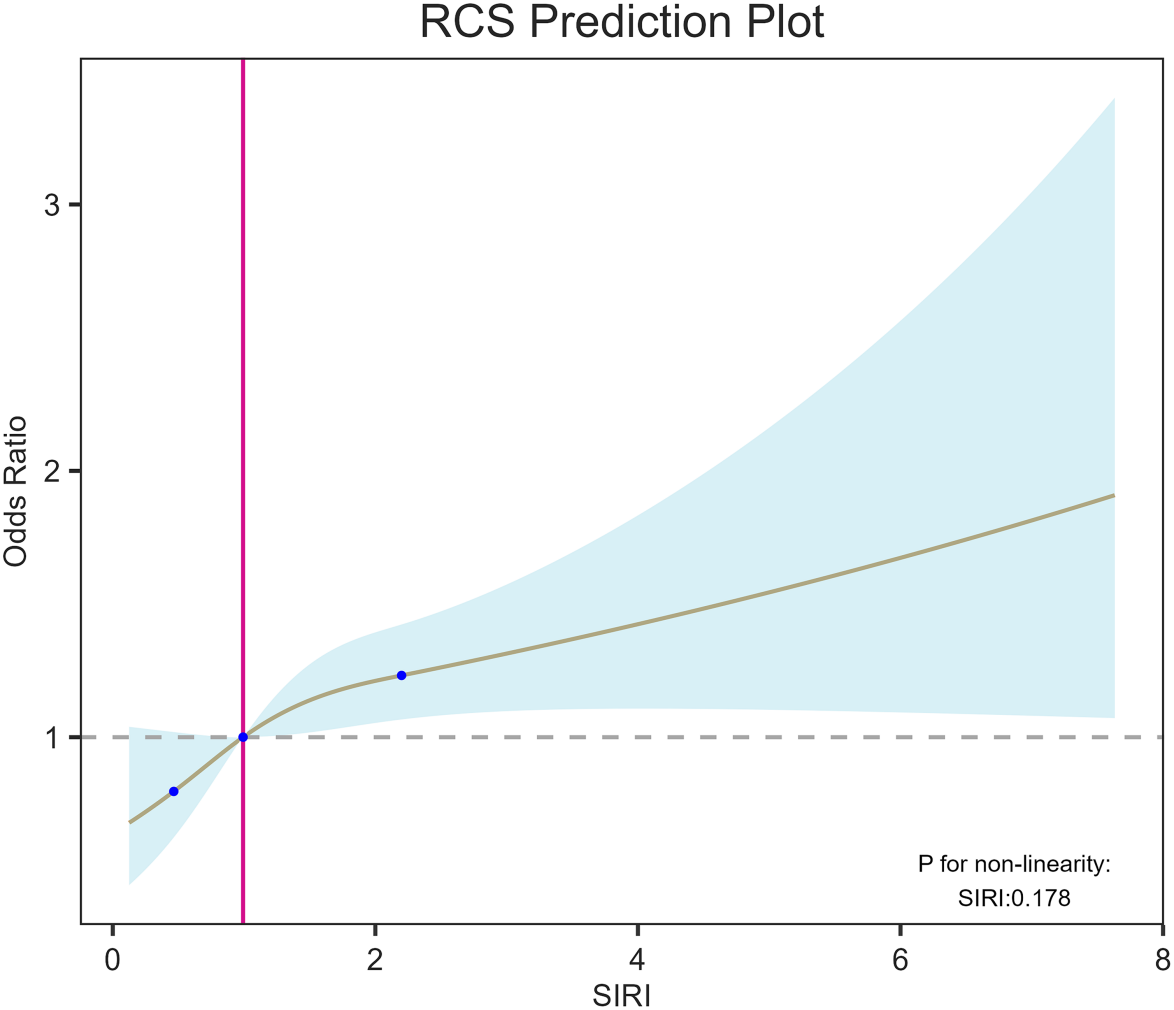
RCS shows a linear relationship between SIRI and coronary heart disease. The fitted regression line is a solid black line; the black dashed line indicates the position where the OR is equal to 1; the shaded area indicates the 95% CI. SIRI, systemic inflammatory response index.

We used RCS to better demonstrate the relationship between SIRI and CHD (Figure 3) and observed a strong linear correlation between SIRI and CHD, we conducted a threshold effect analysis and found an inflection point. After adjusting covariates according to Model 3, the inflection point was 2.86. Observations indicate that when SIRI is below the inflection point, the risk of CHD is lower, when SIRI exceeds the inflection point, the risk increases rapidly.

## 4 Discussion

In this cross-sectional study, we analyzed relevant data from participants aged 40 and older in the NHANES database from 2007 to 2016. The relationship between SIRI and the prevalence of CHD was explored, and we reached the following conclusions: The SIRI of CHD patients was significantly higher than that of non-CHD patients. There was a significant positive correlation between SIRI and CHD, and this relationship persisted after adjusting for multiple confounding factors. After RCS analysis, a significant linear relationship between SIRI and CHD was observed, with a turning point at 2.86. Subgroup analysis and interaction effects indicated a stable relationship between SIRI and CHD across different populations. However, in terms of gender, females were more sensitive to SIRI compared to males.

CHD is a slowly developing chronic disease primarily caused by the progressive narrowing of blood vessels that supply oxygen to the myocardium, with inflammation playing a crucial role in the formation and progression of coronary artery atherosclerosis (21). In clinical practice, complete blood count (CBC) is an easily detectable indicator with rapid result reporting and cost-effectiveness. More importantly, indices and ratios derived from various blood cell counts are relatively reliable inflammatory markers. SIRI integrates monocytes, neutrophils, and lymphocytes, reflecting the status of these three inflammatory cells and providing a more comprehensive assessment of systemic inflammation and immune balance (22). In recent years, SIRI has received increasing attention in predicting the risk of cardiovascular diseases. In a study evaluating the relationship between systemic immune-inflammation index (SII) and systemic inflammation response index (SIRI) with coronary artery disease severity and acute coronary syndrome incidence, 699 patients were included. The results showed that SIRI was associated with diagnosis, with the highest values observed in ACS patients (STEMI), significantly higher than those in stable CAD patients (*P* < 0.01). The highest values of SII and SIRI were observed in patients with three-vessel CAD (23). Li et al. evaluated 959 CAD patients who underwent initial percutaneous coronary intervention, with the primary endpoint being major adverse cardiovascular events and found that elevated SIRI was associated with adverse cardiovascular outcomes in initially diagnosed CAD patients. SIRI can be a simple and practical indicator for identifying high-risk CAD patients after PCI (24). In a cross-sectional study in China, SIRI was recognized as an independent risk factor for CHD in patients with Non-alcoholic Fatty Liver Disease, closely associated with the prediction and severity of CHD (6).

Our study has confirmed the association between SIRI and the risk of CHD. Specifically, elevated SIRI levels increase the risk of CHD, but the underlying mechanisms remain not fully understood. Some studies have suggested that interleukin-1, an inflammatory mediator secreted by neutrophils, promotes abnormal proliferation of vascular smooth muscle cells, leading to early atherosclerosis formation through the induction of endogenous platelet-derived growth factor (25). Additionally, neutrophils release a large number of mediators, including collagenase and elastase, which participate in atherosclerosis, leading to the vulnerability of atherosclerotic plaques (26). Monocytes also play a vital role in the development of CHD. Monocytes are recruited to the intima, where they accumulate, differentiate into macrophages, and further proliferate into foam cells. Foam cells are the main components of atherosclerotic plaques and can promote the formation, development, rupture, and thrombosis of plaques, ultimately leading to myocardial infarction (27). Studies have shown that regulatory T (Treg) cells are a subset of T lymphocytes and play a prominent role in suppressing inflammatory responses (28). Impairment in the number or function of Treg cells can induce plaque formation and progression of atherosclerosis (29).

In subgroup analysis, we found that SIRI was positively correlated with CHD risk in subgroups aged over 60, females, Non-Hispanic Whites, those with high school education or above, obese individuals, drinkers, hypertensive individuals, diabetic and non-angina pectoris individuals. According to interaction tests, we found that the relationship between SIRI and CHD was not influenced by age, race, education level, BMI, smoking status, drinking, hypertension, angina pectoris or diabetes (*P* for interaction > 0.05), suggesting that SIRI may be a reliable indicator for predicting CHD risk in different populations. It is worth noting that with each unit increase in SIRI, the prevalence of CHD in females increases by 33%, suggesting a possible association with declining estrogen levels. Estrogen has extensive and essential physiological effects, not only promoting and maintaining the physiological functions of female reproductive organs and secondary sexual characteristics but also significantly affecting the endocrine, cardiovascular, and metabolic systems, bone growth and maturity, skin, and other aspects (30). The lower incidence of CHD in females compared to males is related to the inhibitory effect of estrogen on the proliferation of vascular smooth muscle cells, affecting the thickness of the vascular wall (31). After menopause, estrogen levels in females decrease significantly, leading to increased infiltration of macrophages and expression levels of inflammatory cytokines such as IL-6, IL-1, and TNF-α (32), which may cause changes in SIRI levels. Furthermore, research has shown that estrogen deficiency can induce calcification of atherosclerotic plaques, and arterial calcification increases with prolonged postmenopausal time. Before 60, vascular calcification in males is twice that of females. Still, after age 60, this gender difference diminishes, indicating the adverse effect of estrogen deficiency on arterial vessels (31). We also found that the prevalence of CHD is higher in obese individuals compared to those with normal weight or overweight. Studies have confirmed that obesity is an independent risk factor for CHD, atrial fibrillation, and heart failure (33). Obesity promotes a series of secondary diseases, including diabetes, insulin resistance, hypertension, dyslipidemia, metabolic syndrome, etc., through various mechanisms such as systemic inflammation, hypercoagulability, and activation of the sympathetic nervous system and renin-angiotensin system, exacerbating cardiovascular diseases (34, 35). It is recommended that obese individuals lose weight, which can effectively reduce the risk of CHD. Moreover, we employed logistic regression to investigate the relationship between SIRI and CHD, discovering a positive correlation between SIRI and CHD in the general population (OR: 1.43, 95% CI: 1.32–1.54, *P* < 0.001). In Model 3, after adjusting for all covariates, we found that SIRI remained significantly associated with CHD risk (OR: 1.12, 95% CI: 1.03–1.22, *P* = 0.008). This model suggests that for every 1 unit increase in SIR, the risk of developing CHD increases by 12%. Based on the RCS, we observed that as SIRI levels increase, the prevalence of CHD also rises. When NLR exceeds 2.86, the risk of CHD increases significantly. The aforementioned research results indicate that SIRI can assist doctors in effectively identifying the risk of CHD, contributing to its prevention and management.

Our study's strength lies in using a large sample provided by the NHANES database, making the statistical results convincing. SIRI is an easily accessible laboratory index that can assist clinicians in identifying high-risk patients with CHD. However, this cross-sectional study also has limitations. For example, cross-sectional studies cannot establish causal relationships between variables. Since the data is collected at a single point in time, it is difficult to determine the temporal sequence of events or whether a specific variable directly influences another variable. The diagnosis of CHD relies on participants’ self-reports rather than diagnoses made by professional physicians, which may introduce bias into the results. Participants in cross-sectional studies may rely on their memory to report past behaviors or experiences. This introduces the possibility of recall bias, where participants may have difficulty accurately remembering or reporting certain information, leading to inaccurate data. Our study results may not be generalizable to populations outside of the NHANES sample. This is due to potential biases and errors in the data collection process, as well as random errors in estimating individual dietary intake (36). Furthermore, although multiple confounding factors were controlled for, unknown confounding factors may still affect the results. We did not include weights when analyzing the data, which provides a more direct interpretation of the results. Considering weights in future studies would better represent the national situation. Finally, this study cannot determine the causal relationship between SIRI and CHD, which requires further research.

## 5 Conclusion

Our findings indicate a significant positive correlation between SIRI and the risk of CHD, especially in female populations. However, current results cannot determine a causal relationship between the two, further prospective studies are needed to confirm their relationship.

## Data Availability

The datasets presented in this study can be found in online repositories. The names of the repository/repositories and accession number(s) can be found in the article/Supplementary Material.
